# Impact of Water Supply Reduction and Cold Storage on Phenolic Compounds from Mango (*Mangifera indica* L. cv. Cogshall) Pulp and Peel

**DOI:** 10.3390/plants11223038

**Published:** 2022-11-10

**Authors:** Rémy Rosalie, Jacques Joas, Christian Mertz, Laurent Dufossé, Mathieu Léchaudel

**Affiliations:** 1 CIRAD, UMR Qualisud, 97410 Saint-Pierre, France; 2 Laboratoire CHEMBIOPRO Chimie et Biotechnologie des Substances Naturelles, Faculté des Sciences et Technologies, Université de La Réunion, 97400 Sainte-Clotilde, France; 3 QualiSud, Université Montpellier, Institut Agro, CIRAD, Avignon Université, Université de la Réunion, 34398 Montpellier, France; 4 Ambiance Fruit, 84250 Le Thor, France; 5 CIRAD, UMR Qualisud, 34398 Montpellier, France; 6 Laboratoire CHEMBIOPRO Chimie et Biotechnologie des Substances Naturelles, Université de La Réunion, ESIROI Agroalimentaire, Parc Technologique, 97490 Sainte-Clotilde, France; 7 CIRAD, UMR QualiSud, Guadeloupe, 97130 Capesterre-Belle-Eau, France

**Keywords:** fruit, health-promoting compounds, mango, flavonoid, tannin

## Abstract

The impacts of water supply reduction and cold storage were investigated on the peels and pulps of cv. Cogshall mangoes, regarding their phenolic compound contents. Phenolics identification was operated using HPLC-MSn for both compartments revealing an unbalanced repartition. Peels had a richer and more complex profile, counting xanthone glycoside (mangiferin), flavonoids (quercetin, kaempferol) and majorly gallotannins. Pulps presented smaller amounts of phenolics and a simpler profile majorly represented by gallotannins and gallic acid derivatives. During fruit ripening, the phenolic contents decreased in both compartments, but faster in the pulp. This behavior can be attributed to the oxidative stress observed in mango pulp during ripening. Cutting down the water supply during the fruit growth triggered an increase in phenolic contents of both the peels and pulp of mango fruits. This increase affected all compounds. Cold storage at 12 or 7 °C led to an increase in mangiferin and flavonoids contents in the fruit peel, interpreted as a stress-response reaction.

## 1. Introduction

Polyphenols are currently the largest group of secondary metabolites [1]. They include some compounds of a low molecular weight, such as simple phenols and phenolic acids, and some compounds of a larger molecular weight, such as xanthones, flavonoids and tannins. Above all, these compounds are a means of physical and chemical defense in plants against biotic or abiotic aggressions [2]. In the event of biotic stress, certain compounds, such as tannins, take part in protecting plants from herbivores due to their strong astringency. Phenolic compounds also play a role in regulating oxidative stress, be it abiotic or biotic, through their antiradical properties enabling them to take on oxidizing or ion-chelating radicals, especially those of transition metals involved in the Haber–Weiss reaction [3,4].

The nutritional benefits of fruits, associated with their bioactive compound contents, are highlighted to promote the consumption of better-quality products. Phenolic compounds are among these molecules of interest and, in the case of mango, some display certain proven biological activities. Mangiferin is found in the bark, leaves, flowers and peel, with a wide action spectrum: antioxidant, anti-cancer, anti-microbial, anti-inflammatory and analgesic properties, among others [5,6]. Flavonoids, which are responsible for the red to violet color of certain varieties and particularly the derivatives of quercetin and kaempferol, exhibit antioxidant, antifungal and anti-cancer activities [7]. 

The impact of growing conditions on phenolic compound synthesis and contents in plants or fruits has been investigated in numerous studies. Water stress in particular has been studied in plants rich in polyphenols of pharmaceutical or nutritional interest. It has been shown that moderate water stress can stimulate phenolic compound accumulation in the aboveground organs of sage (*Salvia officinalis*) [8] or in cumin seeds [9]. The degree of synthesis also depends on the constitutive compartments. For example, the peel in many fruits has a higher content than the pulp because it interfaces with the immediate environment (grapes, peaches, and mangoes). The impact of water stress in the fruits was reported with an increase in polyphenol content, as found in mandarins [10], with stressed peaches displaying higher polyphenol contents than unstressed fruits [11].

Another known stress is the effect of temperature on the concentrations of these metabolites, be it during growth or post-harvest, which highlights the defensive role of phenolic compounds in relation to cold [12]. Refrigeration of fruits after harvesting is usually needed to conserve them, transport them and guarantee that their quality is maintained. Cold storage of several citrus varieties resulted, in most cases, in a significant increase in phenolic compound contents for fruits stored for 16 days at 1 °C then returned to 20 °C for 7 days [13]. A similar tendency was found for peach and nectarine cultivars [14]. Zhao et al. [15] showed that preparatory treatment prior to cold storage (low temperature shock) led to an increase in the phenolic compound content of mango cv. ‘Wacheng’, which was maintained during storage.

The phenolic compound contents of mango can vary depending on the fruit compartment studied [16,17], according to whether the peel, pulp or kernel is considered [18,19]. The pulp, which is the only edible part of the mango, is the least rich, making mango a fruit with a low phenolic compound content [20]. The peel is the most studied compartment of the mango fruit, and most of the bioactive compounds previously mentioned are found in it, including various derivatives of mangiferin, quercetin, kaempferol and condensed gallic acids in the form of tannins [21,22]. The phenolic fraction of the kernel is composed of gallic tannins ranging from tetra to nona-O-galloyl-glucose [22]. The phenolic compound contents of the peel and kernel are approx. between 10 and 100 times richer than pulp. The bioactive compound contents of peel, especially the derivatives of mangiferin and flavonoids, can vary substantially, by a factor of one hundred, between the ‘Tommy Atkins’, ‘Haden’ and ‘Kaew’ cultivars [16,23].

The phenolic compound contents of mango also vary depending on the stage of fruit ripeness [6]. It has been shown in cv. ‘Ataulfo’ that these contents increased initially then decreased in some advanced stages [24,25]. That decrease in polyphenol content in line with ripeness is shared with other species: pomegranates [26], avocados [27], and blueberries [28]. Most studies have focused on the overall quantification of total polyphenols.

Few studies have looked at the impact of abiotic stress conditions on phenolic compound composition and contents taking into account the different compartments of the mango, notably the peel [29,30,31]. This study set out to characterize the composition of the peel and pulp of mangoes cv. ‘Cogshall’, then assess the impact of irrigation conditions and cold storage on the phenolic compound content. In order to achieve highly contrasting situations, the impact of irrigation conditions was assessed by comparing fruits harvested at two stages of ripeness: fruits harvested as green-mature commercial fruits and fruits harvested at the start of ripening on the tree. The effect of storage temperature on the phenolic compound content of ripe fruit was investigated for only one harvesting stage (green-mature stage).

## 2. Results

### 2.1. Identification of the Phenolic Compounds in Mango Peel

**Figure 1:**
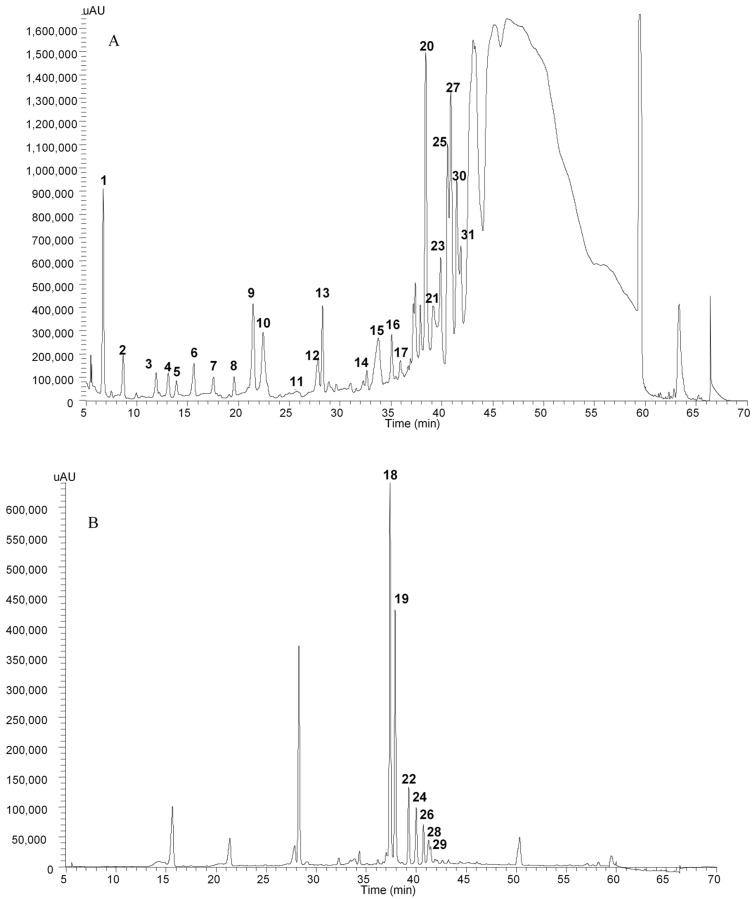
HPLC chromatogram identifying phenolic extracts from the peel of cv. ‘Cogshall’ mango measured at 280 nm (**A**) and 370 nm (**B**).

The separation results obtained for the polyphenols contained in ‘Cogshall’ mango peel are shown in Figure 1. Of the compounds separated, 30 were identified as phenolic compounds (Table 1).

#### 2.1.1. Gallic Acid Derivatives

Compounds **1**, **2**, **16**, **17**, **20**, **21**, **23**, **25**, **27**, **30** and **31** had molecular weights representative of *n*-O-galloyl glycoside with *n* = (4; 6). The fragmentations were separated by one gallate unit (*m*/*z* = 169), lost through ester bond breakage. Some derivatives of galloyl shikimic acid were identified (**4**, **5** and **11**) corresponding to the molar masses (326 g mol^−1^) and fragmentations described by Guimarães et al. [32]. Compounds **6**, **9** and **12** were identified as esters of maclurin glycoside and of mono and di-gallate of maclurin glycoside. 

#### 2.1.2. Xanthone Glycoside

Compound **13** was identified as being mangiferin by its absorption spectrum and its molar mass described by Schieber et al. [33].

#### 2.1.3. Flavonoids

Compound **14** was identified as catechin gallate based on its fragmentation, displaying a loss of gallate grouping, that was obtained in MS_2_ corresponding to that described by Rockenbach et al. [34]. Some derivatives of quercetin were identified and divided between some hexose (**18** and **19**), pentose (**22**, **24** and **26**) rhamnose (**29**) esters identified from their molar masses (464, 434 and 447 g mol^−1^ respectively). Their absorbance spectrum (λ = 355 nm) and their fragmentations correspond to those described in the literature [22,35,36]. A derivative of kaempferol was identified (**28**) as an ester of hexose based on its molecular weight (448 g mol^−1^) and its fragmentation.

#### 2.1.4. Gallotannins

**Figure 2:**
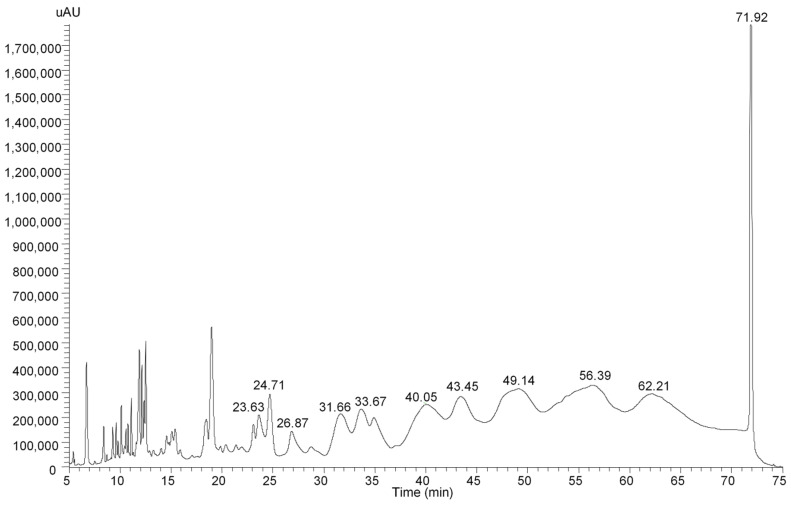
Identification of gallotannins in the peel of cv. ‘Cogshall’ mango measured at 280 nm.

The separation of gallic tannins is shown in Figure 2. The compounds identified were all polymers of gallic acids ranging from hexa to dodeca-O-galloyl glycoside (Table 2). They comprised the majority of compounds contained in the peel.

### 2.2. Quantification of the Phenolic Compounds in Mango Peel

**Figure 3:**
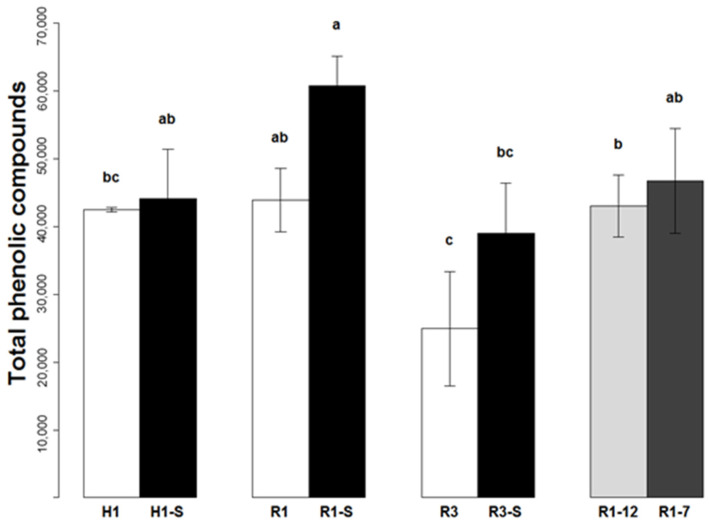
Total phenolic compounds contained in the peel of cv.’Cogshall’ mango (µg g FM^−1^), depending on harvesting dates (H1 and H3 stages), ripening (from H1 = R1 or R1-S, from H3 = R3) and the treatments applied (highest rates of irrigation and non-irrigated (“S”) trees). The white bars indicate the control fruits from the non-irrigated orchard and the grey bars indicate the ripe fruits after 14 days at 12 or 7 °C. Identical letters represent equal mean values (no statistical difference between means at *p* < 0.05 according to Tukey’s multiple comparison test).

The phenolic compound contents of peel are shown in Table 3 and Table 4, and the total contents are shown in Figure 3. The ripeness stage at the time of harvesting had a major effect on the total phenolic compound contents of the mangoes after ripening. The contents of R3 ripe fruits were significantly lower than those measured for R1, whatever the irrigation conditions. The contents for galloyl-O-glycoside, maclurin, galloyl-catechin, gallotannins, quercetin and kaempferol tended to decrease. This decrease was significant for the gallotannins (×0.56). Only the mangiferin content increased in line with the harvesting stage.

The effect of the water deficit on total phenol contents was not visible at the time of harvesting for the fruits harvested at the commercial green-mature stage H1 (Figure 3). However, the fruits subjected to a water deficit had increased contents of maclurin and galloyl-O-glycoside derivatives. That increase was significant for galloyl-O-glycoside derivatives. On the other hand, the gallotannin and galloyl shikimic acid contents were comparable and tended to decrease for galloyl catechin, quercetin and kaempferol, with a significant difference for mangiferin.

After ripening, a significant difference was found for ripe fruits from the non-irrigated treatment, which were richer in phenols than the control fruits, whatever the harvesting stage (R1-S and R3-S fruits). The difference was significant for gallotannins, the major compounds, with an increase of 1.36 times the content of the control fruits. During post-harvest ripening of fruits harvested at the green-mature stage, an increase in the total phenolic compound content was found for fruits from the non-irrigated orchards, whilst it remained stable for the control fruits. A drop in contents for galloyl-O-glycoside derivatives, galloyl shikimic acid and mangiferin was found during the ripening of the control fruits, whilst gallotannin, quercetin, kaempferol, and galloyl catechin contents remained relatively stable. Only the contents for maclurin derivatives increased significantly (×3).

Ripe fruits from a batch subjected to a water deficit displayed a significant increase in contents for gallotannins (×1.44) and mangiferin (×17). A tendency to increase was found for the derivatives of maclurin, galloyl catechin, the derivatives of quercetin and kaempferol. When the fruits were harvested at a later stage (R3-S), a tendency to increase was found overall, and it was significant in the stressed fruits. Only the mangiferin content tended to decrease compared to the control fruits (R3). 

Cold storage had little effect on the total phenolic compound contents in the peel of ripe fruits at 12 and 7 °C (Figure 3). At 12 °C, contents were similar to those of the control fruits for derivatives of galloyl-O-glycosides and gallotannins. Galloyl shikimic acid (×1.53), galloyl catechin and mangiferin tended to increase, whilst the contents for quercetin, derivatives of maclurin and kaempferol decreased significantly. At 7 °C, the galloyl catechin contents tended to increase, and those of mangiferin, derivatives of quercetin and kaempferol clearly increased.

The total phenolic compound contents of ripe fruits were affected by the ripeness stage at the time of harvest, notably through reduction in gallotannin contents. In most cases, the abiotic stress factors applied tended to lead to responses of the phenolic compound metabolism, resulting in an accumulation in the peel. These conditions also led to greater variability in the results, reflected in some larger standard deviations due to varying levels of responses dependent on each fruit.

### 2.3. Quantification of the Phenolic Compounds in Mango Pulp

**Figure 4:**
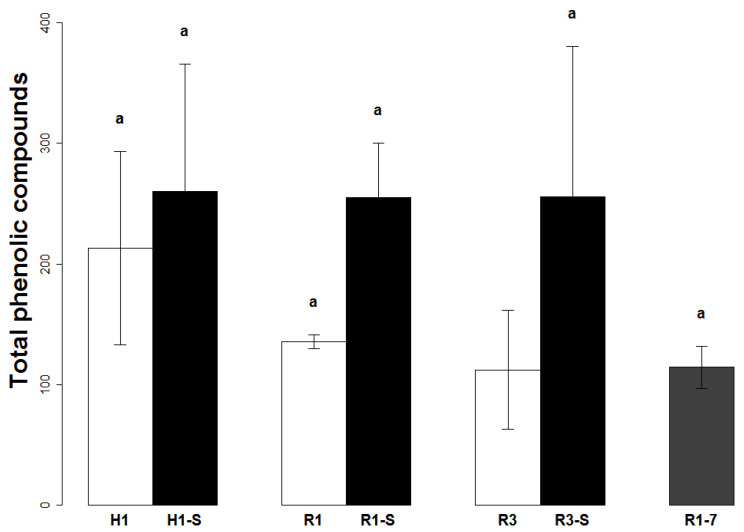
Total phenolic compounds contained in the pulp of cv.’Cogshall’ mango (µg g FM^−1^), depending on harvesting dates (H1 and H3 stages), the treatments applied from control of non-irrigated (“S”) trees, ripening (from H1 = R1 or R1-S, from H3 = R3) and the treatments applied (highest rates of irrigation and non-irrigated (“S”) trees). The white bars indicate the control fruits, the black bars indicate the fruits from the non-irrigated orchard, and the grey bars indicate the ripe fruits after 14 days at 12 or 7 °C. Identical letters represent equal mean values (no statistical difference between means at *p* < 0.05 according to Tukey’s multiple comparison test).

The total phenolic compound contents of ‘Cogshall’ mango pulp were low when compared to those measured in peel (Figure 4, Table 5 and Table 6). The fruits harvested at stage H1 had contents of around 200 µg g^−1^ FM and tended to decrease in line with ripening on the one hand (R1), and with the ripeness stage at harvesting time on the other hand (R3). The water deficit tended to increase phenolic compound accumulation in the pulp, with contents greater than those at harvesting time, even for R3 fruits. Storage at 7 °C had little effect on the total phenolic compound content (Figure 4).

During ripening (H1–R1), the phenolic compound contents generally tended to decrease. The derivatives of gallic acid and maclurin gallate were the cause of that variation, with significant drops of ×0.81 and ×0.58, respectively (Table 5). At maturity (R1–R3), there were no differences in the total content, and only p-coumaric acid displayed a significant decrease.

However, for ripe fruits, the total contents linked to the water deficit tended to increase for all the compounds, and significantly so for the galloyl-O-glycoside and p-coumaric acid contents (Table 5). This tendency, albeit less marked, was also found at stage R3 between the control fruits and stressed fruits.

Lastly, cold storage led to a significant drop in contents for galloyl-O-glycoside, a compound accounting for over 50% of the compounds identified in the pulp, and for p-coumaric acid (Table 6).

## 3. Discussion

The compounds identified in the peel of the ‘Cogshall’ cultivar were similar to those found in the peel of ‘Tommy Atkins’ or Brazilian mangoes [22,23]. The most frequent compounds were derivatives of gallic acid, amounting to 85 to 91% of the area at 280 nm. The ‘Cogshall’ cultivar contained both mangiferin and flavonols, quercetin and kaempferol in its peel. The contents for phenolic derivatives varied from 2.3 to 4.7 mg g FM^−1^, placing the ‘Cogshall’ cultivar between the ‘Tommy Atkins’ and ‘Kent’ cultivars according to the peel values reported by Berardini et al. [16]. The mangiferin and flavonol contents, at around 158 µg g FM^−1^ and 386 µg g FM^−1^, respectively, for the commercial R1 stage, placed Cv ‘Cogshall’ within the mean of the values reported by Barreto et al. [23].

The phenolic compound contents for the pulp of the ‘Cogshall’ cultivar were around 200 times lower than those observed in the peel. This compartmentalization found for Cogshall tallies with the literature [22]. The decrease in phenolic compound contents, in both peel and pulp, during ripening was reported for blueberry by Castrejón et al. [28], where the flavonol, hydroxy-cinnamic acid and overall phenolic compound contents fell, whilst the anthocyanin contents increased to give the blue color to the ripe fruit. Dragovic-Uzelac et al. [37] found a similar behavior in apricot with decreasing flavan-3-ol and flavonol contents. The flavonoid content in the peel of fruits is closely linked to their exposure to UV radiation [38]. Flavonoids play a part in the photo-protection of fruits, and in the attraction of disseminators. The increase in their content is therefore linked to the climatic environment of fruits on the tree [39]. Nevertheless, these compounds are brought into play very little in the case of cv ‘Cogshall’ since their content decreased in line with the ripeness stage at the time of harvesting.

During ripening, fruits are subjected to major oxidative stress linked to an increase in respiratory activity (climacteric rise). The production of reactive oxygen species (ROS) is considerable and leads to molecular and enzymatic defense systems being set in place [40,41]. Phenolic compounds play a major role in maintaining the oxidative balance in fruits, as molecular antioxidants [42]. In mango pulp, although most ROS are dealt with by the enzymatic and ascorbate pathway, the decrease in phenolic compound content found in the pulp of the control fruits (H1-R1), suggested that the antioxidant power of phenolic compounds was effectively being used by the fruit. A drop in phenolic compound content in mango pulp was reported by Kim et al. [24]. However, several studies have reported phenolic compound contents increasing with fruit age [25,43]. The harvesting methods and methods used to determine the physiological age of the fruits lacked reliability in those studies, as they were based either on dates with fruits from different places, or on the degree of peel discoloration estimated visually.

A decrease in gallotannin contents in line with ripeness has also been shown for persimmon cv. ‘Rojo Brillante’ and ‘Kaki Tipo’ [44].

Although the phenolic compound content in peel decreased with the ripeness stage on harvesting, there was in theory no clear relation with ripening since the contents were comparable for the H1-R1 modality followed.

On the other hand, the water deficit induced some quantifiable effects in the peel and pulp during ripening, tending toward an increase for most of the phenolic compounds, with the exception of mangiferin, which did not follow the effect of that stress. A significant increase in total phenolic compound contents was found for peach skin under the effect of a regulated water deficit [11]. On tomatoes, the flavonoid contents measured increased with the water deficit [45]. The impact of this stress was greater for ripe fruits, which would seem to indicate that the compounds accumulated in the skin of the fruits were conserved when the fruits reached maturity. This hypothesis is backed by the reaction of the pulp contents. An accumulation of phenolic compounds in plants subjected to water stress has already been shown for “Cobrançosa” olive subjected to a water deficit, where a significant increase in total phenolic compound contents and phenylalanine ammonia lyase (PAL) activity were measured [46]. In olives, PAL activity is reported to increase in line with the level of water stress applied, which would explain the increase in contents measured here [47].

Cold led to an increase in the overall contents in the peel, primarily linked to the gallotannin content. Cold storage significantly promoted flavonoid and mangiferin synthesis, which tended to increase in line with cold intensity. Flavonoids are acknowledged to display major biological activity as an antioxidant and one of the effects of chilling injury is internal and external browning of the fruits linked, among other things, to the action of polyphenol oxidase (PPO) [48]. The preferential targets of PPO in mango are di- or tri-phenolic nuclei [49] which would make the derivatives of gallic acid, flavonols and flavanols targets for oxidation in the event of membrane breakage. Moreover, an accumulation of flavonoids has been correlated with an MDA, O_2^−^_ and H_2_O_2_ production peak on sugarcane leaves (*Saccharum officinarum* L.) subjected to temperatures of between 4 and 8 °C [50]. In addition, as maclurin is a precursor of mangiferin [51], the clear decrease in the concentration of that compound seen during cold storage must therefore be associated with mangiferin synthesis. Mangiferin, the only xanthone in mango, is proposed as a bioactive compound [52] and would therefore appear to be involved in the peel defense mechanism in response to cold. 

Our results therefore suggest that flavonols and mangiferin in mango peel would seem to be the phenolic compounds most involved in the response to cold.

In the peel, the phenol content of the fruits from modality H1-R1 at 7 °C decreased. This result tallied with the reduction in phenolic compounds found for cv. ‘Wacheng’ mango exposed to cold [15], in parallel with the increase in ROS level, thereby linking the disappearance of phenolic compounds to a reaction to oxidative stress. Although the conditions of this study did not reveal any chilling-injury symptoms, the oxidative stress conditions described in the section (X) show that fruits stored for 14 days at 7 °C placed the R1 stage mangoes under “pre-chilling” conditions corresponding to the initiation of symptoms. Although the response found on peel demonstrated the establishment of a response to oxidative stress, it is trickier to extrapolate in the case of pulp: the phenol concentrations were fairly similar between R1 and R1-7, and the decrease in content would seem, in particular, to reflect the response to ripening stress [53].

## 4. Material and Methods

### 4.1. Crops and Fruits

This study was carried out on mango trees of the ‘Cogshall’ cultivar, grafted onto ‘Maison Rouge’ rootstocks, on the island of Reunion (20°52′48″ S, 55°31′48″ E) at a CIRAD experimental station, 95 m above sea level, during the 2012 season.

#### 4.1.1. Contrasting Irrigation

A protocol varying the degree of irrigation was implemented during the study season (from August 2012 to February 2013). The orchard studied, comprising trees 6 m apart and around 3 m tall, was separated into two sub-orchards: the “control” orchard which received an optimal amount of irrigation, and the “non-irrigated” orchard (abbreviated “S” as stressed fruits), which was deprived of water after flowering. The quantity of water needed for irrigation in the “control” orchard was calculated from the average reference evapotranspiration (ET_0_, [54]) measured in this orchard over the same period in previous years, i.e., around 4 mm. Each day, the volume of water (in m^3^) applied to the mango trees amounted to half of this average ET_0_ multiplied by the area of the orchard (in m^2^). Every 10 days, the actual amount of water acquired for the mango trees was checked using PROBE-w software [55,56], which models the water needs of a plant by calculating the soil–water balance. If the soil water reserve was under 60% of the available water capacity, the volume of water usually applied was doubled for 5 days. The mango trees in the “non-irrigated” orchard only received water from rainfall.

#### 4.1.2. Harvests

The harvests were defined by measuring the variable fluorescence of chlorophyll, Fv, a value that can be linked to the physiological age of fruits when calculated close to the apex, where the first changes in peel color occur [57]. For each season, two ripeness stages were chosen, with Fv values of around 1400 (stage H1) and 950 (stage H3), respectively. Stage H1 corresponded to the commercial green-mature stage. Stage H3 corresponded to the “yellow point “stage (YP) when the apex of the fruit starts to turn yellow, a typical stage indicating the start of the climacteric phase. YP fruits represent the best quality potential for this variety [58]. On harvesting, three stage-H3 fruits were sampled, and six fruits were stored at 20 °C and 90% relative humidity (RH) up to ripening (stage R3).

On harvesting, three stage-H1 fruits were sampled for each of the irrigation treatments. Once harvested, twelve fruits for each treatment were stored at 20 °C and 90% RH up to ripening (stages R1 and R1-S).

### 4.2. Cold Storage

Some of the fruits receiving the highest rates of irrigation were selected to study the effect of cold storage. Thirty-six fruits from the H1 harvest were selected, then separated into three batches. The first batch was stored at 20 °C (12 fruits) for natural and direct ripening to reach a fully ripe stage after about two weeks (stage R1), and the two other batches placed in cold storage for 14 days at 12 °C (12 fruits), or 7 °C (12 fruits), as detailed by Rosalie et al. [59]. The study focused solely on this stage of early maturity, based on the hypothesis that fruits harvested at quite an early green-mature stage are richer in phenols [6] and will in theory show a more marked response than fruits harvested at a more advanced stage of ripeness. At the end of storage, the fruits were inspected visually to ensure there had been no cold impact (any chilling-injury symptoms). They were then transferred to 20 °C and 90% RH for ripening and reached a fully ripe stage after about five days (stages R1, R1-12 and R1-7).

### 4.3. Post-Harvest Measurements and Quality Descriptors

The physiological age of the fruits was assessed by monitoring respiration [60]. The respiratory intensity (RI) of each of the studied fruits (12 fruits per storage condition) was checked daily. RI is expressed by the production of CO_2_ at 20 °C (mmol kg^−1^ h^−1^) in confinement. The fruits in each treatment were placed at 20 °C in sealed 3 L jars. CO_2_ concentration measurements were carried out every 20 min for one hour by gas chromatography using an Agilent M200 device (SRA, Marcy l’Etoile, France), equipped with two manifolds and two columns: Poropak G (8 m), thermostatically controlled at 55 °C, and MS-5A (4 m), thermostatically controlled at 60 °C. Helium and argon were used as the respective carrier gases. All the fruits were sampled 2 days after the respiratory peak, corresponding to their consumption ripeness.

### 4.4. Samples

The fruits were peeled with a knife, taking care to remove minimal pulp, then the pulp was cut into pieces. The peel and pulp of each of the fruits were treated separately in the same way: immersed in liquid nitrogen then ground in a Grindomix grinder (Retsch, Haan, Germany) and lastly stored at −80 °C pending analysis. The mango pulp was then freeze-dried for 48 h at −52 °C. Freeze drying was used to dry part of each sample for analysis. The dried pulp powder was stored in opaque plastic pots with a desiccation capsule at −20 °C.

### 4.5. Extraction of Phenolic Compounds

A total of 0.5 g of plant material (freeze-dried pulp or peel) was taken and homogenized with 20 mL of acetone extraction solvent, methanol: water (50:35:15, *v:v:v*, 0.1% formic acid), and 100 µL of methyl paraben (1 g L^−1^), for 15 min on a linear shaker. The mixture was then centrifuged (6 min, 8000× *g*, 4 °C), then filtered on a Büchner funnel (Watman No. 1). The retentate was extracted with 20 mL of extraction solvent, shaken for 15 min and filtered. The liquid phases were reassembled, evaporated under a partial vacuum and added to 10 mL of methanol. This alcohol solution was filtered at 0.45 µm for injection into high performance liquid chromatography (HPLC).

### 4.6. Identification of Phenolic Compounds

The phenolic compounds of mango pulps and peels were characterized on a Waters 2690 HPLC chromatograph equipped with a Waters 996 DAD detector (Waters Corp., Milford, MA, USA). Separation was achieved using a stationary phase comprising a LiChrospher ODS-2 reversed phase column (Interchim, Montluçon, France) with the dimensions 250 mm × 4.6 mm, 5 µm. The mobile phase consisted of an H_2_O (0.01% HCOOH): CH_3_CN (99.2:0.8, *v*:*v*) mixture (A) and CH_3_CN (B). The analysis of simple phenolic compounds (excluding gallic tannins) was carried out using the following gradient: from 5 to 14% of solvent B in 20 min, a slow increase from 24 to 28% of B for 5 min followed by an 8-min isocratic phase, then an increase from 28 to 32% in 5 min, an increase up to 100% of B in 2 min, maintained at 100% for 5 min to rinse the column of the least polar compounds followed by balancing of B at 5% for 8 min. The gallic tannins contained in the peel were analyzed using a second method: from 5 to 20% of B in 5 min, followed by a 15 min isocratic phase at 20% of B; an increase in 20 to 25% of B for 35 min followed by a 10 min isocratic phase at 25% of B; rinsing with a passage of 25 to 100% of B in 3 min then an isocratic phase at 100% for 5 min; lastly, a return to 5% B in 2 min followed by a balancing phase at 5% for 15 min. The injected volume was 10 µL. After passing through the diode array detector, the flow was divided, and 0.25 mL min^−1^ was sent to the LCQ ion trap mass spectrometer equipped with an electrospray interface (ESI) (Thermo Finnigan, San Jose, CA, USA). The experiments were carried out in negative mode. Desolvation was carried out at 330 °C, spraying at 3500 V. The gas used for desolvation was nitrogen at a flow rate of 70 mL min^−1^. MS/MS and MS3 measurements were carried out with helium as the carrier gas and a collision energy of 25–35 and 50%, respectively. Identifications were obtained based on the molecular ion mass, the MS_n_ data and the visible-UV spectrum.

### 4.7. Quantification of Phenolic Compounds

Phenolic compounds were quantified on a Dionex Ultimate 3000 instrument equipped with a diode array detector (Dionex Ultimate 3000, Dionex Co., Sunnyvale, CA, USA), using a dual mobile phase: A, H_2_O:CH_3_CN (99.8:0.2, 0.01% HCOOH); and B, CH_3_CN (100%). The stationary phase was a Symmetry Shield RP18 reversed-phase column, 250 mm × 4.6 mm × 5 µm, thermostatically controlled at 30 °C with an injection volume of 20 µL. Quantification was based on the identification obtained in HPLC-MS and by comparison with standard curves obtained from commercial gallic acid (CAS No. 149-91-7), mangiferin (CAS No.: 4773-96-0), quercetin (CAS No. 117-39-5), kaempferol (CAS No. 520-18-3) and p-coumaric acid (CAS No. 501-98-4) standards (Sigma Aldrich, St. Louis, MO, USA).

### 4.8. Statistical Analysis

Some analyses of variance were carried out to assess the effect of the treatments and ripeness on all the data presented in the tables and figures. Multiple comparisons between means of measurements at various maturity stages at harvest and after ripening were performed using the Tukey test to evaluate whether or not these means were significantly different (at *p* < 0.05). All the analyses were computed using R software [61] (The R Foundation for Statistical Computing Platform, 2013).

## 5. Conclusions

In view of the phenolic compounds identified in cv. ‘Cogshall’ mango, this cultivar resembles the composition of Florida mangoes (‘Tommy Atkins’, ‘Kent’) [16].

Under stress-free conditions, the effect of fruit ripeness on the phenolic compounds in peel and pulp showed a gradual decrease in line with fruit age, which can be partly linked to oxidative stress increasing as ripening advances toward maturity. Fruits harvested early, but capable of ripening, had a higher phenol content than fruits fully ripe on the tree.

Application of a water deficit during fruit growth led, upon harvesting, to an increase in phenolic compound contents for most of the compounds quantified, compared to the control. This difference remained in both the compartments studied during ripening.

Fruit storage at 12 and 7 °C led to a different reaction between the peel and pulp of the fruits. In peel, the increase in mangiferin and flavonoid contents highlighted a defense reaction in relation to stress. In the pulp of ripe fruits, we can consider that the response to cold was masked by ripening.

The phenolic compound contents and profiles of fruits at harvesting time will depend on both the ripeness stage and irrigation conditions, which will define their evolution during ripening. Concentrations will vary in both peel and pulp. The response to moderate cold will primarily involve the peel.

## Figures and Tables

**Table 1:** Identification of polyphenols in the peel of cv. ‘Cogshall’ mango obtained by HPLC-MSn. nd indicates undetected compound.

Peak No.	Tr (min)	λ max	MS (-)	MS_2_	MS_3_	Identification
1	6.7	281	331	169		galloyl hexose
2	8.6	274	169	125		gallic acid
3	11.9	258, 300	331	169		not identified
4	13.1	278	325	169, 281, 125		galloyl shikimic acid ^a^
5	13.9	278	325	169, 281, 125		galloyl shikimic acid ^a^
6	15.6	290sh, 321	423	333, 303, 193		maclurin 3-C-β-D glycosyl ^b^
7	17.5	298	325	163		*p*-coumarate glycosyl
8	19.5	270	479	443, 281, 237		not identified
9	21.4	276, 325sh	575	423, 303		maclurin mono O galloyl glycosyl ^b^
10	22.4	282	289	245, 205, 179		standard catechin
11	25.7	278	477	325, 169		di-galloyl shikimic acid ^a^
12	27.8	283, 320sh	727	575	485, 405, 313	maclurin di-O-galloyl glycosyl ^b^
13	28.2	260, 321, 369	421	331, 301		Mangiferin ^c^
14	32.6	282	441	289	245, 205, 137	catechin-gallate ^d^
15	33.7	277	nd			nd
16	35.1	280	787	617, 635		tetra O galloyl glycosyl ^b^
17	35.9	280	787	617, 635		tetra O galloyl glycosyl ^b^
18	37.3	256, 357	463	301	179, 151	quercetin O glycosyl ^c^
19	37.9	257, 356	463	301	179, 151	quercetin O glycosyl ^c^
20	38.4	282	939	769, 617		penta O galloyl glycosyl ^b^
21	39.1	280	939	769, 617		penta O galloyl glycosyl ^b^
22	39.2	258, 358	433	301		quercetin pentose ^e^
23	39.8	280	939	769, 617		penta O galloyl glycosyl ^b^
24	40	257, 356	433	301		quercetin pentose ^e^
25	40.5	281	1091			hexa O galloyl glycosyl ^b^
26	40.7	256, 355	433	301		quercetin pentose ^e^
27	40.8	281	1091			hexa O galloyl glycosyl ^b^
28	41.2	268, 346	447	285		kaempferol hexose ^f^
29	41.4	256, 357	447	301		quercetin rhamnoside ^f^
30	41.5	280	1091			hexa O galloyl glycosyl ^b^
31	41.8	280	1091			hexa O galloyl glycosyl ^b^

^a^ [32], ^b^ [22], ^c^ [33], ^d^ [34], ^e^ [35], ^f^ [36].

**Table 2:** Identification of gallic tannins in the peel of cv. ‘Cogshall’ mango obtained by HPLC-MSn.

Tr (min)	λ max	MS/2 (-)	MS (-)	Identification
23.6	280	545	1091	hexa O galloyl glycosyl
24.7	280	545	1091	hexa O galloyl glycosyl
26.8	280	545	1091	hexa O galloyl glycosyl
31.6	280	621	1243	hepta O galloyl glycosyl
33.6	280	621	1243	hepta O galloyl glycosyl
34.9	280	621	1243	hepta O galloyl glycosyl
40	280	697	1395	octo O galloyl glycosyl
43.4	280	697	1395	octo O galloyl glycosyl
47–49.1	280	697, 773	1395, 1547	Octo/nona O galloyl glycosyl
53–57	280	773, 849	1547, 1699	Nona/deca O galloyl glycosyl
61–64	280	849, 925	1699, 1851	Deca/undeca O galloyl glycosyl
72	280	1001		Dodeca O galloyl glycosyl

**Table 3:** Phenolic compound contents in the peel of cv. ‘Cogshall’ mango (µg g FM^−1^) depending on harvest and ripening stages (H1, R1 or H3, R3 stages) and irrigation conditions (highest rates of irrigation and non-irrigated (“S”) trees). Identical letters indicate non-different values (at *p* < 0.05 according to Tukey’s multiple comparison test). Lower case letters indicate the comparisons between control fruits, upper case letters indicate the comparison between fruits from the water deficit treatment. Letters in bold indicate the effect of the harvesting date (R1 R3); the others indicate the effect of ripening (H1 R1). n.s., and * mean that the effect of irrigation treatment is non-significant, and significant at *p* < 0.05, respectively, for each maturity stage.

Peels	*n*	*n*-galloyl-O-glycoside	Derivatives of Maclurin	Galloyl-Shikimic Acid	Galloyl-Catechin	Gallotannins	Mangiferin	Derivatives of Quercetin	Derivatives of Kaempferol
Harvest									
H1	3	2105.17 ± 240.24 a	113.87 ± 16.27 b	120.14 ± 0.5 a	204.43 ± 49.26 a	40027.7 ± 103.27 a	327.65 ± 144.26 a	365.78 ± 96.53 a	16.36 ± 9.17 a
H1-S	3	4428.74 ± 244.97 A	192.94 ± 56.65 A	122.2 ± 2.79 A	165.3 ± 13.47 A	39365.89 ± 7046.38 B	32.36 ± 7.39 B	294.73 ± 61.66 A	*n.d*
Effect		*	*n.s*	*n.s.*	*n.s.*	*n.s.*	*	*n.s*	*-*
Ripe									
R1	3	1782.19 ± 57.08 a **a**	302.44 ± 26.08 a **a**	87.35 ± 30.22 a a	139.51 ± 90.1 a a	41682.98 ± 4646.9 a **a**	157.65 ± 48 a **a**	368.09 ± 27.53 a **a**	18.21 ± 5.03 a **a**
R1-S	3	3116.5 ± 1586.09 A **A**	381.34 ± 132.72 A **A**	130.3 ± 25.73 A **A**	606.04 ± 302.91 A **A**	56810.97 ± 3597.52 A **A**	546.71 ± 327.74 A **A**	434.25 ± 100.4 A **A**	25.87 ± 14.13 A **A**
Effect		*n.s.*	*n.s.*	*n.s.*	*n.s.*	*	*n.s.*	*n.s.*	*n.s.*
R3	4	1412.13 ± 1080.23 **a**	138.13 ± 33.98 **a**	59.62 ± 12.44 a	66.34 ± 29.25 a	23362.8 ± 8763.63 **b**	213.05 ± 14.96 **a**	230.82 ± 108.91 **a**	7.33 ± 2.44 **b**
R3-S	3	1765.1 ± 416.73 **A**	163.39 ± 16.59 **A**	78.56 ± 22.13 **A**	154.54 ± 35.31 **A**	37016.5 ± 6985.17 **B**	198.32 ± 25.98 **A**	412.37 ± 149.42 **A**	27.98 ± 9.52 **A**
Effect		*n.s.*	*n.s.*	*n.s.*	*	*n.s.*	*n.s.*	*n.s.*	*-*

**Table 4:** Phenolic compound contents in the peel of ripe (“R”) cv. ‘Cogshall’ mango (µg g FM^−1^) from H1 harvest depending on storage temperature. n.s., *, ** mean that the effect of storage temperature treatment is non-significant, significant at *p* < 0.05, and *p* < 0.01, respectively, for H1 harvest stage.

Peels	n	*n*-galloyl-O-glycoside	Derivatives of Maclurin	Galloyl-Shikimic Acid	Galloyl-Catechin	Gallotannins	Mangiferin	Derivatives of Quercetin	Derivatives of Kaempferol
R1	3	1782.19 ± 57.08	302.44 ± 26.08	87.35 ± 30.22	139.51 ± 90.1	41682.98 ± 4646.9	157.65 ± 48	368.09 ± 27.53	18.21 ± 5.03
R1-12	4	1717.17 ± 534.22	152.02 ± 52.98	133.23 ± 16.82	186.31 ± 41.51	40903.15 ± 4372.17	191.13 ± 43.53	278.83 ± 35.83	7.25 ± 5.64
R1-7	3	2252.85 ± 723.35	188.63 ± 108.86	137.36 ± 8.31	238.35 ± 38.65	44014.11 ± 7276.26	297.57 ± 43.67	447.09 ± 49.71	26.36 ± 5.63
Effect		*n.s.*	**	*n.s.*	*n.s.*	*n.s.*	*	**	*

**Table 5:** Quantification of phenolic compounds in the pulp of cv. ‘Cogshall’ mango depending on harvest and ripening stages (H1, R1 or H3, R3 stages) and irrigation conditions (highest rates of irrigation and non-irrigated (“S”) trees). Identical letters indicate non-different values. Lowercase letters indicate the comparisons between control fruits, uppercase letters indicate the comparison between fruits from the water deficit treatment. Bold letters indicate the effect of the harvesting date (R1 R3), the others indicate the effect of ripening (H1 R1). n.s., *, and ** mean that the effect of irrigation treatment is non-significant, significant at *p* < 0.05, and *p* < 0.01, respectively, for each maturity stage.

Pulps	*n*-galloyl-O-glycoside	Derivatives of Maclurin	p-Coumaric Acid	Gallotannins
*Harvest*				
H1	90.29 ± 6.73 a	15.39 ± 0.55 a	2.07 ± 0.53 a	105.7 ± 72.96 a
H1-S	103.64 ± 18.67 A	16.27 ± 6.77 A	3.91 ± 0.58 A	136.59 ± 82.76 A
Effect	*n.s*	*n.s*	*	*n.s*
*Ripe*				
R1	73.98 ± 3.16 b **a**	8.9 ± 0.38 b **a**	2.22 ± 0.4 a **a**	50.46 ± 2.7 a **a**
R1-S	94.67 ± 2.22 A **A**	15.75 ± 3.34 A **A**	4.73 ± 1.07 A **A**	140.1 ± 41.34 A **A**
Effect	**	*n.s.*	*	*
R3	42.58 ± 33.45 **a**	9.17 ± 1.84 **a**	*n.d*	60.54 ± 13.88 **a**
R3-S	103.43 ± 20.71 **A**	14.75 ± 2.08 **A**	8.22 ± 2.57 **A**	105.7 ± 72.96 **A**
Effect	***n.s***	*n.s.*	-	*n.s*

**Table 6:** Quantification of phenolic compounds in the pulp of ripe (“R”) cv. ‘Cogshall’ mango from H1 harvest depending on storage temperatures. n.s., and *, mean that the effect of storage temperature treatment is non-significant, and significant at *p* < 0.05, respectively, for H1 harvest stage.

Pulps	*n*-galloyl-O-glycoside	Derivatives of Maclurin	p-Coumaric Acid	Gallotannins
R1	73.98 ± 3.16	8.9 ± 0.38 b	2.22 ± 0.4	50.46 ± 2.7 a
R1-7	61.63 ± 7.29	9.62 ± 1.47	1.09 ± 0.33	42.21 ± 10.67
Effect	*	*n.s*	*	*n.s*

## Data Availability

Not applicable.
